# Corporate political activity of major food companies in Thailand: an assessment and policy recommendations

**DOI:** 10.1186/s12992-018-0432-z

**Published:** 2018-11-22

**Authors:** Nongnuch Jaichuen, Sirinya Phulkerd, Nisachol Certthkrikul, Gary Sacks, Viroj Tangcharoensathien

**Affiliations:** 1 International Health Policy Program (IHPP), Ministry of Public Health, Tiwanon Road, Nonthaburi, 11000 Thailand; 2 Institute for Population and Social Research Mahidol University Salaya, Nakhon Pathom, 73170 Thailand; 3 Deakin University, Geelong, Australia, Global Obesity Centre, 221 Burwood Highway, Burwood, VIC 3125 Australia

**Keywords:** Corporate political activity, Food industry, Policy, Obesity, Non-communicable diseases

## Abstract

**Background:**

The food industry can influence individual and population level food consumption behaviours, shape public preferences and interfere with government policy on obesity prevention and NCDs. This paper identifies the Corporate Political Activity (CPA) of major food companies in Thailand which relate to obesity and NCDs.

**Methods:**

Using the INFORMAS framework to classify CPA, we reviewed publicly available information by 12 food companies between August 2011 and July 2016 in order to identify, analyse and classify the CPA contents. Semi-structured interviews with 17 key stakeholders who are experts in this field supplemented evidence from the document review. Data analysis applied a thematic approach.

**Results:**

Food industry in Thailand applied a variety of CPA strategies and practices. The two most common strategies were constituency building and information and messaging.

**Conclusion:**

The diverse range of CPA strategies which influence government policy and public opinion can undermine efforts to prevent obesity and diet-related NCDs. We recommend systematic monitoring of their CPA, strengthening mechanisms to hold the food industry accountable for their role in protecting and promoting the nutrition and health of the population, introducing mandatory registration of lobbyists, mandatory disclosure of political donations, and stronger oversight of conflicts of interest among the government actors.

**Electronic supplementary material:**

The online version of this article (10.1186/s12992-018-0432-z) contains supplementary material, which is available to authorized users.

## Background

At the global level, obesity is a major public health problem [1]. While rates of obesity have leveled off in high-income countries [2], in middle-income countries such as Thailand [3, 4] rates are increasing alarmingly. Food environments play a significant role in influencing diet and consumption behavior and are significant contributors to obesity and diet -related non-communicable diseases (NCDs) [5, 6].

The food industry plays an important role in shaping food environments and food choices. Food companies have high potential to produce a healthy food supply, but the processed food sub-sector has been criticised for driving people to desire high-calorie foods and become conditioned overeaters and for creating food environments that promote overconsumption [7]. Evidence shows that, globally, the processed food industry is active to ensure that policy and regulatory environments are structured in their favour, and that they use their power and influence over political processes to minimize the criticisms and health concerns associated with their products [8, 9]. The strategies and tactics used are referred to as Corporate Political Activity (CPA), and have been shown to be similar in nature to the CPA used by tobacco and alcohol companies [10–12].

Food industry CPA includes, among other tactics, framing the debate and shaping the evidence on diet to mould public preference in ways that favour their products, establishing relationships with policy makers, and seeking community support in various ways [13, 14].

Food and beverage is one of Thailand’s largest manufacturing industries, with a diverse size and profile, ranging from large multinational companies to domestic operators. Thailand, one of the emerging economies, has the third largest market of snack foods and non-alcoholic beverages in the South East Asia region after Indonesia and Vietnam [15]. Globally, Thailand ranks 19th in the world’s snack food market and 25th in the beverages market [16].

Despite growing concern over the influence and interference of the food industry on government policies that affect health of the population, there is limited evidence on the strategies and practices used to influence these policies in low and middle income countries. In response to commitments to tackle NCD and obesity challenges, this paper identified and analyzed the CPA used by major food companies in Thailand which may have negative impacts on government policy addressing obesity and diet -related NCDs.

## Methods

The study drew upon an approach which identifies the CPAs in the food industry, proposed by INFORMAS (International Network for Food and Obesity /non-communicable Diseases Research, Monitoring and Action Support) [17]. The approach suggests two synergistic qualitative methods: a document review of publicly available information on CPAs; and semi-structured interviews with key stakeholders involved in diet- and public health-related policies. The approach categorizes commonly used CPA [17] into six groups: a) information and messaging, b) financial incentive, c) constituency building, d) legal strategies, e) policy substitution, and f) opposition fragmentation and de-stabilisation.

### Selection of companies

There are two-stage processes in selecting food and beverage companies for data collection. First, a list of most prominent companies for each type of products was developed using Thai market share data from the 2015 Euromonitor Passport database. Second, a Thai Expert Advisory Group (TEAG) was constituted to select food companies from the listing based on the following criteria: include a diversity of food categories, include companies with the largest market share overall and in major food categories, the diversity of company’s policies and practices on nutrition and health and the likelihood of gaining access to their CPA.

Members of the TEAG consisted of eight health and non-health experts from governmental and non-governmental sectors specifically, two senior government officials, three university professors and three leaders of non-governmental organizations.

Of the initial list of 20 food and beverage companies across all categories, twelve were selected by the TEAG to be included in the study. Five companies that pledged self-regulating market promotion to children also known as the Thai Pledge, and one sugar company that played a significant role in sugary foods and beverages were purposively selected.

Of these twelve companies, nine sold packaged food and the others sold soft drinks, fast food and sugar (Table 1). Packaged food companies were further categorised as 1) baked, biscuit and snack bars (BBS); 2) confectionery, ice cream and frozen desserts (CIF); 3) ready meals (RM); and 4) sweet and savoury snacks (SSS). Half of the companies were Thai owned; the remaining companies were transnational.

**Table 1:** Characteristics of twelve selected companies

No	Company	Market share (% of total market share in its food sector/category)	Food sector (food category)	Type of ownership
*Packaged foods - Baked, biscuit and snack bars (BBS)* ^*b*^				
1	President Bakery PCL	28.5	Packaged food (Baked goods)	Thai
2	Thai President Foods Public Co Ltd	8.5	Packaged food (Biscuit and snack bars)	Thai
*Packaged foods - Confectionery, ice cream and frozen desserts (CIF)* ^*c*^				
3	Mars Inc^a^	9.8	Packaged food (Confectionery)	Transnational company
4	Unilever Group^a^	60.3	Packaged food (Ice cream and frozen desserts)	Transnational company
5	Nestle^a^	13.4	Packaged food (Ice cream and frozen desserts)	Transnational company
*Packaged foods - Ready meals(RM)* ^*d*^				
6	Charoen Pokphand Group [CP]	40.6	Ready meals	Thai
*Packaged foods - Sweet and savoury snacks (SSS)*				
7	PepsiCo Inc^a^	24.1	Packaged food (Sweet and savory snacks)	Transnational company
8	BerliJucker Plc [BJC]	9.7	Packaged food (Sweet and savory snacks)	Thai
9	TaoKaeNoi Food & Marketing Co Ltd	8.2	Packaged food (Sweet and savory snacks)	Thai
*Soft drinks*				
10	CocaCola Co^a^	28.0	Soft drinks	Transnational company
*Fast food restaurant*				
11	Yum! Brands Inc	13.3	Fast food	Transnational company
*Sugar supplier*				
12	Mitr Phol group	20.3	Sugar	Thai

^a^Committed to self-regulating marketing for children under Thai Pledge policy

^b^Example products in this category include baked, biscuit and snack bars

^c^Example products in this category include confectionery, ice cream and frozen desserts

^d^Example products in this category include ready meals

### Document review

Company documents and information related to company CPAs were collected from various publicly available data sources, as recommended by INFORMAS [17] and with guidance from TEAG. Data sources included company websites, domestic and international social media, websites of other relevant private sector organisations such as the Federation of Thai Industry. Databases included library databases at major universities in Thailand, the Matichon newspaper database (the largest newspaper database in Thailand). Websites and published materials from relevant Ministries and Departments were also reviewed as well as those of other organisations including political parties and the Thai Health Promotion Foundation. Data reviewed covers the period between Aug 2011 and July 2016. Information was obtained in either Thai or English, or both, depending on the source.

### Semi-structured interviews of key stakeholders

After completing the document review, semi-structured interviews with key stakeholders were conducted to supplement evidence from the document review. Stakeholders were those who were involved in policy processes on obesity prevention and diet-related NCDs, or experts who had several years experiences and significant expertise in monitoring or researching private sector organization policies and practices across different public health areas such as food and nutrition, alcohol, tobacco, breastfeeding and other public health related fields, or experts who could provide insight and first-hand experience on the CPA strategies and tactics used by food companies. A list of stakeholders was initially drawn from secondary data sources, including government websites and documents, NGO publications and websites, major Thai newspapers and Internet searches, as well the recommendations from TEAG. Snowball sampling was then applied to identify other relevant stakeholders until information became saturated.

A list of 23 multi-sectoral stakeholders were drawn and invited but only 17 agreed to participate. Of these, four were from governmental organizations (GO) who were either former or current senior officials in Ministries or Departments; four were from not-for-profit organisations (NGO); three were from academia (AC); and six were from private sector organisations (PV) including food companies, industry associations and media companies. Interviews took place between October and November 2016.

Pilot interview questions (see Additional file 1) were tested and revised prior to data collection. Written informed consent was sought before face-to-face interviews and tape recordings held at either the stakeholder’s workplace or wherever was most convenient. Interviews lasted approximately one hour. Field notes were taken by researchers during each interview. Confidentiality was fully observed.

### Data analysis

The CPA data from the document review and interviews were analysed using a qualitative thematic inductive approach with reference to the CPA framework [17]. Contents in the interview transcripts were analysed where emerging themes were identified. Relevant quotations in Thai which supported a theme were translated to English, and no attributes of the stakeholder were identified except for their stakeholder group affiliation. In this study, data triangulation involved the comparison between the qualitative data derived from semi-structured interviews with stakeholders, and the contents of the document review from various publicly available data sources. Where significant differences in findings from the two sources were found, these were noted and discussed in the text.

The analysis was completed by three independent researchers in a two-step process. The first and second researchers analysed contents from desk-review documents and interview scripts independently, then discussed to reach the final themes. Discrepancies between the two researchers were arbitrated by the third researcher and through consensus among the three.

This study was approved by the Research Ethics Board of the Institute for Development of Human Research Protection, Thailand in November 23, 2016.

## Results

Although all six CPA strategies were used, two most common strategies emerged, constituency building (1031 out of the total 1563 documents analysed) and information and messaging (529 documents). Certain practices such as lobbying and stressing the economic importance of the industry as part of the information and messaging strategy, the legal strategy, and the strategy on opposition, fragmentation and destabilization were identified through interviews with key informants. The ready meals; baked, biscuit and snack bars; and soft drink companies were the most active in CPA.

Additional file 2 identified contents extracted from the document review, where four CPA strategies and nine practices were commonly applied by these twelve companies. The CPA content analysis from stakeholder interviews and the document review are summarised in Table 2.

**Table 2:** Summary content analysis of CPA strategies and practices commonly applied by 12 selected companies; from document reviews and stakeholder interviews

Strategies and practices of CPA		Total contents related applied by CPA
Six Strategies	16 Practices	
1 Information and messaging	1.1 Lobby policy makers ^a^	0
	1.2 Stress the economic importance of the industry ^a^	0
	1.3 Promote deregulation ^b^	1
	1.4 Frame the debate on diet- and public health-related issues^b^	264
	1.5 Shape the evidence base on diet and public health-related issues ^b, c^	264
2 Financial incentive	2.1 Fund and provide financial incentives to political parties and policymakers ^b^	1
3 Constituency building	3.1 Establish relationships with key opinion leaders and health organizations ^b^	63
	3.2 Seek involvement with the community ^b^	570
	3.3 Establish relationships with policymakers ^b, c^	356
	3.4 Establish relationships with the media ^b, c^	42
4 Legal strategies	4.1 Use legal action (or the threat thereof) against public policies or opponents ^a^	0
	4.2 Influence the development of trade and investment agreements ^a^	0
5 Policy substitution	5.1 Develop and promote alternatives to policies ^b^	1
6 Opposition fragmentation and destabilisation	6.1 Criticise public health advocates	0
	6.2 Create multiple voices against public health measures ^a^	0
	6.3 Infiltrate, monitor and distract public health advocates, groups and organisations	0
Total number of CPA practices identified		1562

^a^From stakeholder interviews only

^b^From both document review and stakeholder interviews

^C^New mechanisms identified specific to Thai context

### Strategy one: Information and messaging

This is the second most common CPA strategy, consisting mainly of shaping evidence using various techniques and framing messages in ways that look positive to the food industry.

1. Lobbying

Stakeholder interviews with key informants from government and NGOs identified lobbying as a common practice. The lobbying usually goes against government efforts to prevent obesity and diet-related NCDs.

“The food industry knew that we [Ministry of Public Health] planned to introduce a policy using traffic light system in food labeling. They [the industry] completely went against our plan. …It [name of the transnational company] contacted me to discuss this policy. In parallel, they approached the politicians. [Name of policy regulator] tried to compromise with private sectors while NGOs and academia convened several public forums, where debates were held around traffic light labeling”. (GO) said at the end, the traffic light nutrition labeling policy was defeated.

2. Stress the economic importance of the industry

Although the document review did not find evidence that the food industry applies this in their CPA practices, in the semi-structured interviews, participants from academic organisations and government organisations reported that industry frequently referred to the economic importance of the industry in discussions with government and the media. The academic representatives saw this framing a barrier to public health efforts to prevent and control NCDs.

“…it [food industry] continues stressing this [their economic importance] as always. As the government perceives that the private sector has a major role in boosting the national economy, it is more supportive of and sympathetic towards them than other sectors.” (AC)

The academic’s view was consistent with another GO:

“This [solving health problems] can be seen from two perspectives: economic aspect and social and health. People in the economic sector always have a louder voice than the social and health sector. They [public health people] keep talking about protecting consumers, [but] it doesn’t help those [business owners] to earn more from selling their goods.” (GO)

3. Promote deregulation

The document review and interviews captured the promotion of deregulation, notably by the sugar company complaining about job losses in manufacturing and agricultural (sugar cane) sectors, and the increased administrative burden to the company [DocumentA1 in the Additional file 2]. NGO participants in the interview recalled the situations when the sugar company applied this practice, in counteracting sugar-sweetened beverage taxation, (SBB tax) proposals by experts and NGOs to the government.

“Food industry representatives always claimed that this policy [SBB Tax] would have a huge negative impact on sugar-cane farmers, as the [sugar] production would drop and then farm employment would decrease.”(NGO)

4. Frame the debate on diet- and public health-related issues

Review of documents and contents from the interviews indicated that many food industry sectors apply this strategy. One fast food company stressed the good traits of the company as that the rice used by all restaurants is clean, safe and free from contamination, comes from suppliers who passed quality assurance standards such as Good Manufacturing Practice, Hazard Analysis and Critical Control Points [Document A2 in Additional file 2]. A packaged food company emphasized its good intentions in addressing public health related issues. Reviews of the company website showed that their “Healthy Kids Global Programme” focuses on nutrition education and physical activity, providing information on balanced diets, positive approaches to food and practical advice on improving eating habits [Document A3].

A tactic was applied to shift the blame away from their products, and frame the social debates towards the awareness of the consumers, responsible eating behaviours and health concerns.

“I think if consumers have knowledge, they can make their own healthy food choices. If they know that product is unhealthy or harmful to their health, they shouldn’t choose it.” (PV)

5. Shape the evidence based on diet and public health related issues

Review of documents revealed that the most frequently used techniques were to support funding research to academics and research institutions; paying experts to speak or promote research evidence that favoured the company; and using data that favoured the company to promote the public image. The company also sponsored educational activities, such as seminars in schools, universities and NGOs. An example of a specific activity in this area is when the sugar company signed a memorandum of understanding with one major Thai university which provided a research grant for the development of food products to improve oral health [DocumentA4]. One transnational food company stated on their website that it conducted seminars on ‘pre-school children nutrition’ and invited: the Directors of Departments and Divisions on nutrition from the universities as their speakers [DocumentA5]. Annual reports of some packaged food companies revealed the names of university professors who are experts on food and nutrition as the company’s [paid] member of their advisory board and consultants [DocumentA6]. Incentivizing experts to conduct research or produce evidence in favour of the food industry is a synergy of two combined strategies - information and messaging and financial incentives.

Participant interviews confirmed evidence identified from the document review on research funding and sponsoring education to academics and research institutions.

“If you ask us [food company] do we want to do research? I just say ‘No’. We do not have time to do it… our result is not acceptable to the public because we [the industry] have many conflicts of interest. So... we fund the research to other research agencies.” (PV)

### Strategy two: Financial incentives

We define financial incentives as the food industry directly fund or offer financial incentives either in cash or in kind such as donations, paying for entertainment or other financial support to political parties and policy makers.

1. Fund and provide financial incentives to political parties and policymakers

The document review of the media, confirmed by interviews, revealed that the ‘ready meal’ packaged food sector uses financial incentives through donations to policy makers and political parties [DocumentA7]. Interviewees raised concerns that such practices impeded success in introducing government policies to prevent obesity and NCDs.

“The true story behind this failure [introduce policy on traffic light food labeling] was that [name of political party] needed money to support the party’s activities.” (GO)

“Although the government [name of responsible agency] had tried to improve the food labeling [using the traffic light system], it was not successful. The food company is backed by the political party that received money from them.” (NGO)

Importantly, it should be noted that many participants did not feel comfortable about discussing the use of financial incentives, despite the assurance of their confidentiality by researchers.

### Strategy three: Constituency building

Constituency building is the most common CPA strategy. This was revealed through document reviews and confirmed by interviews. Practices under this strategy such as seeking involvement from the community and establishing relationships with policymakers were the most frequently documented. There are four practices under this strategy:

1. Establish relationships with key opinion leaders and health organizations

Clearly, the document review showed that all food industry sectors had established relationships with key opinion leaders. For example, the website of one company noted that:

“During the [2011] severe flood in Bangkok and several provinces in the central region, the [BBS group] company cooperated with the World Instant Noodles Association (WINA) to assist the flood victims via the [Royal] Foundation [a Foundation that serves to provide prompt, timely and necessary responses to problems affecting the Thai people through various development projects]” [DocumentA8].

A company reported to have established strong links with a charitable foundation:

“The [RM] company has donated food to the Foundation [a Foundation that aims to promote honesty and constancy of the monarch, public health, sport and doing research for human development, established by former Prime Minister of Thailand] continuously since 2006”[DocumentA9].

2. Seek involvement and relationships with the community

The document review as confirmed by interviews, indicated that all five food industry sectors applied this CPA practice. For example, some packaged food and soft drink companies sought involvement in the community by creating giving programs to donate their products and money for disabled groups and people in underserved areas. Every year, a BBS group company provides scholarships and financial aid to students, students with disabilities and patients [DocumentA10]. A company in the CIF group gives all profits from selling some boxed meals to the Border Patrol schools [DocumentA11] and a soft drink company provides lunch for orphanages [DocumentA12].

In addition, the document review found that the food industry provided support for physical activity initiatives and events for children and communities, targeting children and school settings. This included “Charity Bowling” [DocumentA13], “A walk rally to promote protection against osteoporosis” [DocumentA14], “Junior Football Championship” [DocumentA15] and “Painting Contest to celebrate Mother’s Day” [DocumentA16]. Examples of community events include “Cycle Club” [Document A17], a voluntary camp for “Volunteers for Rural Development” [DocumentA18], “Making Hard and Transparent Bar Soaps Training Course” for people in the community [DocumentA19], and a fast food company’s “Add Hope” for customers to donate money for disadvantaged people [DocumentA20].

Interviews also identified the presence of industry sponsored programs in schools:

“We provide them [schools] healthy foods like our low-calorie rice noodle products during [their] sport events.” (PV)

3. Establish relationships with policymakers

Review of documents showed that the food industry fully engaged in public-private interactions. For example, one BBS company donated 200,000 Baht (~USD 5800) to a 2011 co-project between Thailand’s Senate and the Royal Thai Army for flood victims in order to relieve suffering from flooding [DocumentA21].

Establishing informal relationships with Thai and international policy makers were applied. This included a senior-level representative of a company in the BBS group having a chance to accompany Deputy Minister of Commerce and Director of Department of Export Promotion to support the mission of the Ministry of Commerce. In this case, to promote exportation of Thai products [DocumentA22]. The RM company welcomed the Russian Federation Ambassador to the Kingdom of Thailand and conferred of him the Federation’s investment in Thailand [DocumentA23]. The president of a fast food company gave a book on ‘Taking people with you’ focused on business management and human resources to the ambassador of the United States of America to Thailand at the US Fair 2012″[DocumentA24].

There were also documented instances of formal relationships between senior members of the food industry and senior government positions. For example, a report from one BBS company stated that, “the Director of the company is a Member of the National Legislative Assembly, Economy, Commerce and Industry Committee and Vice-President of the Chamber of Commerce at the same time” [Document A25].

Some participants perceived that these relationships between food company personnel and government agencies led to the involvement of the industry in policy processes as technical supporters or advisors to policymakers. Participants noted the widespread involvement of food industry representatives in government committees.

“At present, every national committee has industry representatives, such as representatives of the Federation of Thai Industries, the chamber of commerce and related associations. They are sitting on the national committee, namely the food safety, food security and food and nutrition related health committee. The company provides information and works together with officials.” (PV)

“Fortunately, we have a chance to work with [name of government agency] so when they have any policy plan, they will inform us. We have been working with them since they started [developing policy]. For example, the agency invited me to provide information and asked for my opinion. Therefore, we had an opportunity to give our people information and give them [government] feedback whether this [policy] should be implemented or if there were any other better options.” (PV)

There was also evidence of a revolving door, a situation in which a senior government official changes jobs to a senior position in the food industry or vice versa. Participants mentioned that this could benefit the food industry in terms of knowing government practices and policy processes, or representing industry interests within government. Examples identified from the document review included a former senator, a member of the National Legislative Assembly, a former Deputy Prime Minister, a former Minister, a former Director General of a relevant government Department and a former advisor to the Prime Minister. In addition, an AC participant identified that:

“There is a politician [name of politician] and another one - former director of [name of government agency] - sitting on a food company’s board.” (AC)

Some food companies were also found to develop relationships with the government through relatives of individuals who work in government agencies or political parties. A report from one ready meal company stated that a cousin-in-law of the president of [ready meal company] was former Ministry of Commerce, member of the parliament in 2011 and politician in a certain political party [DocumentA26].

4. Establish relationships with the media

Establishing relationships with the media refers to actions by the food industry to develop close relationships with media agencies, journalists and other bloggers to facilitate media advocacy in favour of the food industry. These actions go beyond activities that would typically be considered public relations, and potentially include paying individual journalists to protect their corporate image and interests, and aiming to influence editorial decisions. As the media is a powerful instrument to communicate and shape public views, this strategy is applied, although it is less common compared with building relationships with communities and policy makers.

The study identified this CPA practice both through document review and interviews. The evidence revealed that the food industry established relationships with media organizations and journalists to facilitate media advocacy. Examples include a ready meal company which organized a thank you party for a media agency, called “thank you party ‘you are my hero’”[DocumentA27] and the CEO and President of one company gave a gift basket to one of Management Committee members of a Thai daily newspaper company [DocumentA28]. One transnational soft drinks company gave drinking water to a Thai daily newspaper company [DocumentA29] and the marketing manager of a fast food company gave their food products to the editorial department of a Thai daily newspaper company [Document A30].

In addition, one NGO participant identified that there were instances of food companies paying individual journalists to protect company corporate images and interests as part of their reporting.

“Food companies gave journalists money every month to keep them in their control.” (NGO)

Clearly, this CPA practice has demonstrated the industry’s tactics in integrating financial incentives (strategy two) as an entry point for constituency building (strategy three) such as establishing links and relationship with the opinion leaders, the communities, policy makers and the media.

### Strategy four: Legal strategies

The use of the ‘legal strategy’ was revealed by experts in the interviews. Such practices include the use of legal action, posing legal threats against public policies or the industry’s opponents, or influence over the development of trade and investment agreements.

Interviews with participants indicated that the food industry threatened to litigate against potential government policy through legal channels provided by the World Trade Organization (WTO). In these cases, participants indicated that the industry aimed to intimidate policy makers by citing potential barriers to free trade if such policy was introduced.

“The food industry has its own way to restrict policy [traffic light labeling]. They didn’t talk much here [with the government] but they can bring the case to the WTO court [in such a way to threaten the policy makers].” (NGO).

### Strategy five: Policy substitution

#### Develop and promote alternatives to policies

Document review and interviews identified the use of a policy substitution strategy though it was not commonly applied. The industry developed and promoted their own alternative policies or competed with government policies. For example, voluntary reformulation of certain products [Document A31, 32, 33] such as producing better nutrient-balanced food, low-sugar and low-salt products. Also, some of these companies pledged to self-regulate unhealthy food marketing to children and supported the government in promoting healthier food choices. The food industry also stressed its preference for non-legal binding national policy instruments, which they saw as the most effective way to prevent obesity and NCDs.

“The food industry disagreed with the government to use laws to solve problems. I feel that the legal system should be the last resort after having proved all other tools are ineffective. Further, collaboration between government and the private sector was the most effective way since the industry is closer to consumers than government.” (PV)

### Strategy six: Opposition fragmentation and destabilisation

There are three practices of opposition fragmentation and destabilisation strategy. These include criticising public health advocates, creating multiple voices against public health measures and infiltrate, monitor and distract public health advocates, groups and organisations. One practice was found in this strategy:

#### Create multiple voices against public health measures

No food companies in this study applied the opposition fragmentation and destabilisation strategy as identified through the document review; however, participants in the semi-structured interviews mentioned that the industry created opposition to public health policies. One participant commented that the industry established proxy organisations to act behind-the-scenes to protect the interests of the company. This tactic is similar to the pharmaceutical industries in using puppet agencies [18].

“The food industry establishes a proxy association or club to act against government policy through submission of petition letter to government protesting actions which may affect the industry.” (GO)

## Discussion

This study examined the CPA strategies applied by twelve major food companies in Thailand, using two sources of data: publicly available information and interviews with key stakeholders. The study showed that companies applied several sophisticated practices, which could shape public policy and programs in their favour. The two most commonly used CPA strategies were constituency building and information and messaging. These CPA strategies were used across different sectors of the industry, whether national or transnational corporations. The food industry uses combined CPA strategies and practices to achieve their goals in influencing policies. For example, on front-of-pack traffic light system nutrition labelling, they applied the information and messaging strategy, by lobbying policy makers against the policy and advocating for deregulation. They also used the financial incentive strategy, particularly by funding political parties and policy makers, as well as the legal strategy, threatening to bring the case to WTO Court.

While most information on CPAs was publicly accessible, some was discovered following interviews with stakeholders. For example, there was no publicly available information on lobbying policy makers, although interviews revealed that it was used against the traffic light food labelling and the government proposal was defeated. While high-income countries such as the United States, UK and Canada have lobbyist registries with information on lobbyists, the hiring companies and lobbying issues either for or against [19–21], no such registration systems exist in Thailand. Moreover, declaration of donations to political parties in Thailand is scarce in the absence of legislation. Political parties which receive donations are required to declare them but enforcement is poor.

Common CPA strategies used by the food industry in Thailand are similar to those in Australia [13] and Fiji [14], such as information and messaging and constituency building, which suggests the food industry applies a standard repertoire of tactics. Transnational corporation experiences [22] are replicated or modified by national corporations. This study also identified country-specific CPA practices, which were also practiced in other countries. They included the use of public-private partnerships (PPPs); provision of industry-sponsored education to health professionals; relatives working in government agencies or political parties to bridge relationships; industry sponsoring of individual journalists to boost company image and protect its interests in media coverage; and the inclusion of specific clauses in company contracts with suppliers to protect against potential unfavourable government regulations. In the US, Australia and Canada the food industry seeks to influence individual journalists on how their businesses are portrayed in the media. For example, some journalists were frequently contacted by restaurants inviting them to dinner and sometimes offering to pay for their visit [23].

The application of two or more strategies magnifies the impacts of CPAs. For example, using financial incentives to sponsor academia to generate biased evidence to frame the debates in favour of the industry and using financial incentives to establish relationships with the media to formulate social discourse focused on individual food choices and responsibility of their own health. Further study is required to identify the inter-links and combined effects of multiple strategies.

CPA using PPPs has become common. For example, in the area of food reformulation the US, UK and Canada all have high-profile PPPs with extensive food industry participation [24, 25]. Though some evidence indicates the positive outcome of PPP in terms of improved food systems [26–28], others have raised concerns about the effectiveness [29] and significant associated risks of PPP [30].

CPA findings from this study are more or less similar to strategies and practices used by other industries, such as the alcohol and tobacco industries. A large body of evidence confirms that CPA tactics are common across corporations and industries [31] [32]. In particular, those establishing close relationships with health professionals and their associations, lobbying governments to relax regulations, advocating voluntary self-regulation and distorting research findings [8, 33]. Findings from this study are also consistent with previous Thai studies on CPA applied by the alcohol and tobacco industries [34–38].

The food industry often plays an active role in public health efforts to control NCDs [28, 33, 39] in order to protect their businesses, sales volumes and profit margins. Often public health goals contradict the attempt to maximise and pay high dividends to investors [33]. Evidence shows that although food companies have modified product formulas and voluntarily promised to produce more nutritious products, they have continued to produce and promote the consumption of less nutritious versions [8, 40]. Another study identified the gaps between the promises made by processed food companies and their actual practices, suggesting that voluntary mechanisms alone do not work and that parallel effective regulations by government are required [40].

The strength of this study is a combination of document review of publicly available information from various data sources and insights from interviews with key stakeholders. To our knowledge, it is the first time such a study has been conducted in Thailand and in South East Asia. This data is essential in assisting the Thai government and stakeholders and audiences outside Thailand in developing evidence informed policies and interventions to prevent people from eating unhealthy food and ultimately protect the health of populations.

Despite the strengths of this study, a few limitations were identified. Public information on CPAs only covers the last 6 years. Moreover, CPA with its potential negative image is well hidden. CPA activities related to lobbying, opposition fragmentation and destabilisation are concealed and never documented in public reports except donations to communities which boost the company’s public image.

Critically, this study explicitly adopted a public health perspective in considering the CPA utilised in the food industry. Other perspectives, such as corporate or economic perspectives, are likely to result in different classifications of the industry practices identified in this study. For example, many practices in this study cited under the constituency building strategy could be considered as part of the social responsibility activities carried out by businesses. It is acknowledged that certain actions of food companies include aspects that could be classified under multiple practices or strategies. For example, financial contributions to political parties clearly involve the financial incentives strategy but are also likely to be targeted at building relationships with policy makers, which falls under the constituency building strategy. In these cases, actions were classified under the strategy where they were deemed to fit best.

Although key informant interviews were useful as supplementing secondary data analysis, the content is subjective by nature, difficult to verify, and also unlikely to represent a comprehensive picture of food industry activities unless a very large sample of stakeholders are covered. In the Thai context, participants - despite assurance of confidentiality - did not feel comfortable discussing all aspects of industry influence such as funding and provision of financial incentives to political parties, academia and policymakers.

A government is generally held accountable to protect and promote the health of the people. Governments in high-income countries, and even more so in low- and middle-income countries have been assessed as having a low response to, or belated recognition of health and nutritional threats from the food industry [28, 41]. Policy decisions can be distorted by the discourse generated by the food industry when they argue that choices regarding healthy foods, moderate eating habits and adequate physical activities which prevent obesity are key, hence education of consumers is required instead of regulation of industry.

This study demonstrates that given the increased prevalence of obesity and food-related NCDs combined with unregulated CPA, urgent policy and actions are required. A few suggestions have been provided below.

Establish and sustain a CPA monitoring system as a foundation and entry point for evidence-based policy development. Either the government, non-government organizations, or research agencies should host such regular monitoring systems.

Establish legislation and enforcement of a mandatory lobby register with regular reporting containing the name and profile of the hiring and the lobby agencies. Reporting should include the issues, means, mechanisms and the persons or agencies being lobbied. Also enforced should be the mandatory reporting of donations to political parties, individuals, and journalists. The Journalist Council should tighten the implementation of the code of conduct and sanctions for code violations.

Prevent government officials from receiving financial incentives from the food industry. This is seen as interfering with the function and discharge of their duties to protect the health of populations and appropriate legal sanctions.

Strengthen the role of independent civil society organisations operating as watchdogs in monitoring CPA. This should involve monitoring academia which generates biased evidence, journalists who shape public opinion in the food industry’s favour, and government officials who are influenced by the food industry. This will help improve the accountability of governments and the industry itself in relation to nutrition and population health [42, 43].

## Conclusions

In response to a growing concern about the impact of the food industry on unhealthy food environments and their influence on government policies, this is the first study in Thailand which presents comprehensive evidence about the strategies and practices used by the food industry. The food industry employs a variety and combination of CPA strategies to increase their power and influence over government policies. The approaches are similar to those used by food companies outside Thailand and by other industries such as tobacco and alcohol. A monitoring system for CPA is recommended to identify entry points for action, in parallel with regulation and enforcement to ensure transparency and hold all partners accountable. This includes the food industry, academia, journalists, media organisations and the government.

## Additional files

Additional file 1: Description of CPA strategies. (PDF 449 kb)

Additional file 2: Supporting Information [Documents retrieved during data collection]. (PDF 391 kb)
