# Supplementary material for: Corporate political activity of major food companies in Thailand: an assessment and policy recommendations
Source: Global Health. 2018 Nov 22;14:115. doi: 10.1186/s12992-018-0432-z (PMC6249932; doi:10.1186/s12992-018-0432-z)
Supplement: Supplementary file 1 — Description of CPA strategies. (PDF 449 kb) [file 12992_2018_432_MOESM1_ESM.pdf]

Additional file 1: Description of CPA strategies

CPA: corporate political activity of the food industry		
**Strategies**	**Practices**	**Mechanisms**
1.Information and messaging	1.1 Lobby policy makers	1.Lobby directly and indirectly (through third parties) to influence legislation and regulation so that it is favorable to the industry
	1.2 Stress the economic importance of the industry	1. Stress the number of jobs supported and the money generated for the economy
	1.3 Promote deregulation	1. Highlight the potential burden associated with regulation (losses of jobs, administrative burden)
		2. Demonize the 'nanny state'
		3. Threaten to withdraw investments if new public health policies are introduced
	1.4 Frame the debate on diet- and public health-related issues	1. Shift the blame away from the food industry, e.g. focus on individual responsibility
		2. Promote the good intentions and stress the good traits of the food industry
		3. Emphasize the food industry's actions to address public health-related issues
	1.5 Shape the evidence base on diet and public health-related issues	1. Fund research, including through academics, research institutions and front groups
		2. Pay scientists as advisers, consultants or spokespersons
		3. Cherry pick data that favors the industry
		4. Disseminate and use non-peer reviewed or unpublished evidence
		5. Participate in and host scientific events
		6. Provide industry-sponsored education*
		7. Suppress or influence the dissemination of research
		8. Emphasize disagreement among scientists and focus on doubt in science
		9. Criticize evidence and emphasize its complexity and uncertainty

CPA: corporate political activity of the food industry		
**Strategies**	**Practices**	**Mechanisms**
2.Financial incentive	2.1 Fund and provide financial incentives to political parties and policymakers	1. Provide donations, gifts, entertainment or other financial inducements
3.Constituency building	3.1 Establish relationships with key opinion leaders and health organizations	1. Promote public-private interactions, including philanthropic, transactional and transformational relationships
		2. Support professional organizations through funding and/or advertising in their publications
		3. Establish informal relationships with key opinion leaders
	3.2 Seek involvement in the community	1. Undertake corporate philanthropy
		2. Support physical activity initiatives
		3. Support events (such as for youth or the arts) and community-level initiatives
	3.3 Establish relationships with policymakers	1. Seek involvement in working groups, technical groups and advisory groups
		2. Provide technical support and advice to policymakers
		3. Use the 'revolving door', e.g. ex-food industry staff work in government organizations and vice versa
		4. Use relatives who work in government or political party*
		5. Participate in public-private interactions, including philanthropic, transactional and transformational relationships*
	3.4 Establish relationships with the media	1. Establish close relationships with media organizations, journalists and bloggers to facilitate media advocacy
		2. Pay for individual journalists to protect their cooperate image and interest*
4.Legal	4.1 Use legal action (or the threat thereof) against public policies or opponents	1. Litigate or threaten to litigate against governments, organizations or individuals

CPA: corporate political activity of the food industry		
**Strategies**	**Practices**	**Mechanisms**
	4.2 Influence the development of trade and investment agreements	1. Influence the development of trade and investment agreements such that clauses favorable to the industry are included (e.g. limited trade restrictions, mechanisms for corporations to sue governments)
5.Policy substitution	5.1 Develop and promote alternatives to policies	1. Develop and promote voluntary codes, self-regulation and non-regulatory initiatives
6.Opposition fragmentation and destabilization	6.1 Criticize public health advocates	1. Criticize public health advocates personally and publicly, e.g. through the media, blogs
	6.2 Create multiple voices against public health measures	1. Establish fake grassroots organizations ('astroturfing')
		2. Procure the support of community and business groups to oppose public health measures
	6.3 Infiltrate, monitor and distract public health advocates, groups and organizations	1. Monitor the operations and advocacy strategies of public health advocates, groups and organizations
		2. Support the placement of industry-friendly personnel within health organizations

*New mechanisms identified specific to Thai context
