# Supplementary material for: Corporate political activity of major food companies in Thailand: an assessment and policy recommendations
Source: Global Health. 2018 Nov 22;14:115. doi: 10.1186/s12992-018-0432-z (PMC6249932; doi:10.1186/s12992-018-0432-z)
Supplement: Supplementary file 2 — Supporting Information [Documents retrieved during data collection]. (PDF 391 kb) [file 12992_2018_432_MOESM2_ESM.pdf]

# Additional file 2: Four strategies and nine practices for CPA commonly applied by the twelve selected food industries identified from reviews of documents

| Reference in manuscript | Food industry actor | Source | Strategy | Practice (code used for analysis) | Data coded | Source of evidence: website URL |
|---|---|---|---|---|---|---|
| A1 | MirtPhol | Newspaper | Information and messaging | Promote deregulation | “Komkrich Nakalak is a MirtPhol company representative. He requests to postpone increasing SSB tax because he is afraid this policy has effects on customers, farmers and their businesses.” | Ban Muang newspaper; on Jul 23, 2016 |
| A2 | Yum! Thailand [KFC] | Newspaper | Information and messaging | Frame the debate on diet- and public health-related issues | “The rice used at every KFC restaurant is clean, safe and free from contamination, sourced from suppliers who have passed recognized standards such as Good Manufacturing Practice, Hazard Analysis and Critical Control Points, BRC.” | Nation newspaper; Jul 27, 2013 |
| A3 | Nestle | Company website | Information and messaging | Frame the debate on diet- and public health-related issues | “We aim to help children understand the role that nutrition plays in their lives and how to balance between good nutrition and active lifestyle. The Nestle Healthy Kids Global Programme focuses on nutrition education and physical activity, providing information on balanced diets, positive approaches to food and practical advice on improving eating habits. Promote healthy diets and lifestyles, including physical activity” | company website [on Sep 28, 2016] http://www.nestle.com/csv/nutrition/healthy-diet<br>CSV report 2015, on page 12 |
| A4 | MirtPhol | Company website and newspaper | Information and messaging | Shape the evidence base on diet and public health-related issues | “Dean of Faculty of Dentistry, Mahidol University signed MOU with MitrPhol representative to improve product that help oral health.” | company website [on Oct 3, 2016] http://www.mitrphol.com/index.php/th/news_and_activity/newsdetail/98<br>Bangkok Business newspaper; on May 9, 2016 |
| A5 | Nestle | Newspaper | Information and messaging | Shape the evidence base on diet and public health | “Nestle conducted seminar “pre-school child nutrition”. The speaker is Head of Nutrition, from the department of paediatrics, Chulalongkorn University” | Daily New newspaper; on Jul 27, 2016 |
| A6 | Thai President Foods | Company website | Information and messaging | Shape the evidence base on diet and public health | “Professor Dr. Visith Chavasit is chairman of risk management committee and Professor of the Institute of Nutrition in Mahidol University” | Annual report 2015, on page 248 |

| Reference in manuscript | Food industry actor | Source | Strategy | Practice (code used for analysis) | Data coded | Source of evidence: website URL |
|---|---|---|---|---|---|---|
| A7 | Charoen Pokphand Group [CP] | Newspaper | Financial incentive | Fund and provide financial incentives to political parties and policymakers | "Democrat party got 65.9 million baht donation from Chearavanont Family..." | Post Today online; on Sep 10, 2016 http://www.posttoday.com/analysis/91492. |
| A8 | Thai President Foods | Company website | Constituency building | Establish relationships with key opinion leaders and health organizations | "During the severe flood in Bangkok and several provinces in the Central part of Thailand, the Company donated Mama (instant noodle) products to the victims via many government and private agents. For example, the Company offered Mama Cup to Thairat Newspaper and "Kan Pak" TV program to fill the rescue package for disaster victim around the country, and cooperated with World Instant Noodles Association (WINA) to assist the victim via Chai Pattana Foundation and the Royal Thai Navy." | Annual report 2011, on page 34 |
| A9 | CP | Newspaper | Constituency building | Establish relationships with key opinion leaders and health organizations | "'Generous Hearts for the South' Project, operated by the General Prem Tinsulanonda Foundation established by Former Prime Minister and Statesman Prem Tinsulanonda. This project focuses on youths in five Southern provinces: Pattanee, Yala, Naratiwat, Satun, and Songkhla. The Company has donated food to the Foundation continuously since 2006." | Opt-news newspaper; on Jun 6, 2011 |
| A10 | President Foods | Company website | Constituency building | Seek involvement in the community | "Donate Company's products and money to help Home for Multiple-Handicapped Blind Children, Ramintra, Bangkok" | Annual report 2014, on page 239 |
| A11 | Unilever Thailand | Facebook | Constituency building | Seek involvement in the community | "All income from meal box would be donated to l Border Patrol school." | https://www.facebook.com/Unileverthai/ [on Aug 29, 2016] |
| A12 | Coca-Cola Thailand [Haadtip] | Company website | Constituency building | Seek involvement in the community | "Provided lunch for orphanages in Ban Nakorn Sri Thammarat, Nakorn Sri Thammarat" | Annual report, on page 35 |

| Reference in manuscript | Food industry actor | Source | Strategy | Practice (code used for analysis) | Data coded | Source of evidence: website URL |
|---|---|---|---|---|---|---|
| A13 | President Foods | Company website | Constituency building | Seek involvement in the community | "Farmhouse sponsored the King Mongkut's University of Technology North Bangkok (KMUTNB) Charity Bowling 2016" | company website [on August 3, 2016] http://www.farmhouse.co.th/article/detail/267 |
| A14 | BerliJucker [BJC] | Company website | Constituency building | Seek involvement in the community | "National food company supported a walk rally to promote protection against osteoporosis for the 2nd year mark the International Osteoporosis" [BJC:] | CSR Report 2013, on page 9 |
| A15 | Coca-Cola Thailand [Haadtip] | Company website | Constituency building | Seek involvement in the community | "Sponsored the 16th Coke Cup Thailand Junior Football Championship" | Annual report, on page 11 |
| A16 | Thai President Foods | Company website | Constituency building | Seek involvement in the community | "Sponsoring Painting Contest of "18th Arts Represent Love with National food company [Mama]" to celebrate Mother Day" | Annual report 2011, on page 35 |
| A17 | CP | Newspaper | Constituency building | Seek involvement in the community | CP Food (CPF) established "CPF Cycle Club" | 5 newspapers<br>Thansettakij; onApr 24, 2014<br>Thai Post; on Apr 22, 2014<br>Matichol;on Apr 22, 2014<br>Khaosod; on Apr 21, 2014<br>Prachachat Business; on Apr 17, 2014 |
| A18 | CP | Newspaper | Constituency building | Seek involvement in the community | "The Company established its own voluntary camp, "CPF Volunteers for Rural Development" in 2011, with a view to calling upon youths and young adults from all educational institutions in Thailand to volunteer in remote, poverty-ridden rural areas. To date, 10 such summer camps for volunteer-students from 20 educational institutions have been implemented." | Banmuang newspaper; on Oct 3, 2011 |

| Reference in manuscript | Food industry actor | Source | Strategy | Practice (code used for analysis) | Data coded | Source of evidence: website URL |
|---|---|---|---|---|---|---|
| A19 | BJC | Company website | Constituency building | Seek involvement in the community | "Staffs of Rubia Industry Co.,Ltd conducted a training course on how to make clear soap for the community" | CSR Report 2013, on page 12 |
| A20 | Yum! Thailand [KFC] | Newspaper | Constituency building | Seek involvement in the community | KFC develop an initiative to support community, called "KFC add hope" | PrachachatBusiness; Jan18 2016 |
| A21 | President Foods | Company website | Constituency building | Establish relationships with policymakers | The company donated 200,000 Baht to the co-project between The Senate and Royal Thai Army for flood victims in order to relief suffering occurring to the flood victims. General Theeradej Meepian, Speaker of The Senate received such donation. | Annual report 2011[on page 39] http://www.farmhouse.co.th/documents/investor/th/Annual_Report_11.pdf [August 2, 2016] |
| A22 | Thai President Foods | Newspaper | Constituency building | Establish relationships with policymakers | "Chief Executive Officer of national food company has a chance to go with Deputy Minister of Commerce and Director General Department of Export Promotion to India in order to support the mission of Ministry of Commerce " | Thansettakij newspaper; on Aug 23, 2012 |
| A23 | CP | Newspaper | Constituency building | Establish relationships with policymakers | "CPF welcomed ambassador of the Embassy of the Russian Federation to the Kingdom of Thailand and conferred with him about investment in his country" | 3 newspapers<br>Thai Post; on Dec 30, 2014<br>Matichol; on Dec 24, 2014<br>Kaohoon newspaper; on Dec 23, 20014 |
| A24 | Yum! Thailand | Company website | Constituency building | Establish relationships with policymakers | "President of Yum Restaurants International (Thailand) Co., Ltd. who own KFC and Pizza Hut restaurant in Thailand gave "Taking people with you" business management and human resource book to ambassador of the Embassy of the United State of America to the Kingdom of Thailand at US Fair 2012" | Company website [on Sep 28, 2016] http://www.yum.co.th/th/media_center_details.php?id=9 |

| Reference in manuscript | Food industry actor | Source | Strategy | Practice (code used for analysis) | Data coded | Source of evidence: website URL |
|---|---|---|---|---|---|---|
| A25 | Thai President Foods | Websites of the company and Senate | Constituency building | Establish relationships with policymakers | "CEO of company is appointed as Member of the National Legislative Assembly No.74, representing the Economy, Commerce and Industry Committee and also served as Vice-President of the Chamber of Commerce" | Annual report 2015,on page 250 http://www.senate.go.th/w3c/senate/secretariat.php |
| A26 | CP | Newspaper | Constituency building | Establish relationships with policymakers | "Cousin in law of the president of CP Group was former Minister of Ministry of Commerce and member of the parliament in 2011. Now, he is a politician in Pheu Thai Party. | Isra news and Thairath newspaper online http://www.isranews.org/investigate/item/15828-2012-08-18-08-17-02.html?pop=1&tmpl=component&print=1, On Sep 10,2016 http://www.thairath.co.th/person/8677, On Sep 10,2016 |
| A27 | CP | Newspaper | Constituency building | Establish relationships with the media | CPF organized party for thank you to Mass Media, called Thank you party "you are my hero". | Kaohoon newspaper; on Nov 29,2013 |
| A28 | BJC | Newspaper | Constituency building | Establish relationships with the media | "CEO and President of BJC gave gift basket to Thakul Boonpan, Matichol newspaper." | Matichol newspaper; on Jan 22, 2015 |
| A29 | Coca-Cola Thailand [Thai Nam Tip] | Newspaper | Constituency building | Establish relationships with the media | "Coca Cola Thailand and Thai Nam Tip Co., Ltd gave drinking water to Matichol group." | Matichol newspaper; on Nov 9, 2011 |
| A30 | Yum! Thailand [KFC] | Newspaper | Constituency building | Establish relationships with the media | "KFC marketing manager of Yums restaurant International (Thailand) Co., Ltd gave KFC fried chicken to Khaosod editorial department." | Khaosod newspaper; on Aug 8, 2015 |

| Reference in manuscript | Food industry actor | Source | Strategy | Practice (code used for analysis) | Data coded | Source of evidence: website URL |
|---|---|---|---|---|---|---|
| A31 | Thai President Foods | Websites of the company | Policy substitution | Develop and promote alternatives to policies | Even though the quantity of Sodium in "MAMA" product is not different from other type of noodles, Thai President Foods PCL joined the statement of "Earth Food" in the 7th meeting of World Instant Noodles Association in 2010, held in Malaysia, and urged global instant noodles manufacturers to reduce their Sodium ingredient by 5% every year. | Annual report 2012 [on page 31] |
| A32 | CP | Websites of the company | Policy substitution | Develop and promote alternatives to policies | The company has launched the "CP Balance" product line offering healthy instant food products that provide balance-nutrition and are high in fibre and vitamins and low in cholesterol, saturated fat, and sodium. | Health Nutrition policy, on page 4 |
| A33 | Unilever Thailand | Websites of the company | Policy substitution | Develop and promote alternatives to policies | "We've reduced sugar in beverages, particular tea products since 2010 and our goal is to continue to reduce sugar by 2020." | Company website https://www.unilever.co.th/sustainable-living/introducing-our-plan/improving-nutrition<br>Newspaper<br>Post Today; on July 29, 2016 |
